# Delusions and Responsibility for Action: Insights from the Breivik Case

**DOI:** 10.1007/s12152-013-9198-4

**Published:** 2013-12-19

**Authors:** Lisa Bortolotti, Matthew R. Broome, Matteo Mameli

**Affiliations:** 1 Philosophy Department, University of Birmingham, Edgbaston, B15 2TT UK; 2 Department of Psychiatry, University of Oxford, Oxford, UK; 3 Department of Philosophy, King’s College London, London, UK

**Keywords:** Delusions, Moral responsibility, Criminal insanity

## Abstract

What factors should be taken into account when attributing criminal responsibility to perpetrators of severe crimes? We discuss the Breivik case, and the considerations which led to holding Breivik accountable for his criminal acts. We put some pressure on the view that experiencing certain psychiatric symptoms or receiving a certain psychiatric diagnosis is sufficient to establish criminal insanity. We also argue that the presence of delusional beliefs, often regarded as a key factor in determining responsibility, is neither necessary nor sufficient for criminal insanity.

## Crime and Psychiatric Diagnosis

Has the Breivik case taught us anything about the relationship between psychiatric diagnosis and responsibility for criminal action? In July 2011, Anders Breivik killed 77 people in Norway. In August 2012, he was sentenced to 21 years in prison. As part of his first psychiatric evaluation, conducted by Torgeir Husby and Synne Sørheim, he was diagnosed with paranoid schizophrenia and some of his most implausible beliefs were regarded as persistent, systematised, bizarre delusions. For instance, one belief he reported is that he was the leader of a Knights Templar organisation which, according to the Norwegian police, does not actually exist.

If it had been shown that Breivik experienced psychotic symptoms at the time of his crime, then he would have faced trial with a diagnosis of psychosis, and he would not have been regarded as accountable for his actions. This is because, in the Norwegian Criminal Procedure Code, when one has psychotic symptoms, one cannot be attributed criminal responsibility for action: “a person is not criminally accountable if psychotic, unconscious, or severely mentally retarded at the time of the crime” ([1], page 17). For the Norwegian Code, what psychosis involves is determined by the current diagnostic manuals, and in DSM-IV it was defined as the presence of hallucination or delusions; in the wider definition, bizarre behaviour and speech were also included. If Breivik’s diagnosis of a psychotic disorder had been confirmed based on his symptoms, he would have been regarded as “criminally insane” and sentenced to compulsory psychiatric treatment.

The Norwegian court system employs the biological principle, which means that the presence of psychosis at the time a crime is committed will automatically result in a ruling of insanity, independent of the intent of the perpetrator. Any justified doubt in this regard should favour insanity. ([2], p. 2413)

However, this first assessment leading to the diagnosis of schizophrenia was overruled by a second assessment, conducted by Agnar Aspaas and Terje Tørrissen, according to which Breivik’s strange beliefs were not psychotic symptoms in the context of schizophrenia or of some other psychotic disorder, but could be explained by a personality disorder. Based on the fact that he never manifested hallucinations or Schneiderian first-rank symptoms, this second pair of assessors rejected the diagnosis of schizophrenia previously given to Breivik and described his behaviour as caused by a narcissistic personality disorder accompanied by pathological lying. Phenomena that had been interpreted as negative symptoms of schizophrenia in the earlier assessment (emotional disturbances, indifference, social withdrawal) were given a different explanation [1]. As a result of the second assessment and his new diagnosis, Breivik was held accountable for his actions because he was thought not to have been psychotic at the time of his criminal act. In Norwegian Law the connection between psychiatric symptoms and attributions of responsibility is very direct: if one is found to have had psychotic symptoms at the time of one’s criminal acts, one is not held responsible for those acts.

Some have suggested that the Breivik case is not dissimilar from the case of David Copeland, known as the London Nail Bomber, who killed 3 people and injured 139 by using homemade nail bombs in a series of attacks in April 1999. At Copeland’s trial, experts were also divided over whether he should be given a diagnosis of schizophrenia or of personality disorder [3]. He pleaded guilty to manslaughter on the grounds of diminished responsibility, but he was convicted of murder and given six concurrent life sentences.

In English Law, the McNaughton Rules specify when one is not to be held responsible for one’s actions due to mental illness. Such rules rely on one not knowing the quality or nature of the act, not knowing that it was wrong, and being under an “insane delusion” that prevents the appreciation of the true nature or quality of the act. This is only an admissible defence in cases of murder. Given the fairly high threshold, in homicide cases, the diminished responsibility defence is often used instead. For the diminished responsibility defence to apply, it is not sufficient to demonstrate that there is an abnormality of mind. This abnormality must be due to development, injury, or illness, and must impair responsibility for actions “substantially”, as judged by individual medical experts and juries. In schizophrenia, for example, we know that, at a group level, there are cognitive and neuropsychological impairments, even when patients are remitted and taking medication. However, it is not always easy to establish without further testing whether such impairments affect a given individual, and if so, whether they interfere with that individual’s local, particular, and context-specific decisions in a “substantial” way.

John Gunn [4], one of the psychiatric experts at the Copeland trial, argues that there was no doubt that Copeland had severe schizophrenia (a diagnosis also confirmed by the team at the Hospital where Copeland had previously received treatment), but the court still favoured the view that Copeland was responsible for his crimes.

On many occasions due deference is given by lawyers and jurymen to medical opinion, thus conferring apparent power to psychiatrists. This is an illusion because the power is on loan and can be withdrawn when the politics of a case, usually a high profile case, demand it. The mental-abnormality excuse used to mitigate many crimes of homicide is not available for cases deemed inexcusable by the newspapers, politicians and public opinion. If by some skilful advocacy an ‘inexcusable’ crime is excused, then a public outcry occurs after the trial. ([4], page 62)

Why did Breivik’s psychiatric evaluation change? [5] Were there similar pressures as those identified by Gunn in the Copeland case? Some feel that the psychiatrists responsible for the first evaluation made a mistake and they did not take into account the right-wing context in which Breivik’s assertions were made [2]. As a result, the first assessors emphasised the implausibility and idiosyncrasy of Breivik’s beliefs.

Another interpretation of the case, favoured by Ingrid Melle [1], is that Breivik had schizophrenia all along, but his symptoms were less florid at the time of the second interview, which occurred several months after the crime. The psychiatrists involved in his second assessment found that Breivik had taken distance from many of his wildest claims – for instance, he himself suggested that he just *wanted to believe that* he had played a leading role in the Knights Templar organisation. To support this reading, one could suggest that a change of diagnosis on the basis that Breivik did not have hallucinations or Schneiderian first-rank symptoms is problematic, as these symptoms are not necessary for a diagnosis of schizophrenia. Moreover, people with narcissistic personality disorder do not experience any psychotic symptoms, unless some other comorbid problem is also present. Further, one delusion was enough for a diagnosis of psychosis in DSM-IV, even if not for a diagnosis of schizophrenia (for instance, one could have a psychotic disorder such as delusional disorder or an affective psychosis), which means that if Breivik had any delusions at all, then a diagnosis of psychosis would have been legitimate.

There is a tension in the reaction of the public to murderers such as Copeland and Breivik. On the one hand, there is often a tendency to think that the killer “must be mad” to commit such ominous crimes. On the other hand, there is an overwhelming desire for retribution. The killer “must be punished” in the appropriate way, and psychiatric treatment is not perceived as a sufficient response to severe crimes. Retributivist intuitions and the desire to punish are strong and extremely resistant to change. In cases of emotionally shocking crimes they easily override the intuitions that (a) horrendous crimes must be the consequence of some sort of mental malfunctioning, and that (b) people with mental illness may not be morally responsible for the crimes they commit. When Simon Wessely commented upon the Breivik case in the *Lancet*, he exposed two common misconceptions about psychiatry. The first is that “outrageous crimes must mean mental illness”. The second is that “the purpose of psychiatry is to get people off” ([6], page 1563). As Wessely suggests, these positions are badly supported by evidence, blind us to the important details of individual cases, and lead to excessively polarised debates on mental health and moral and legal responsibility for action.

Interpretations of the changes in diagnosis between Breivik’s first and second psychiatric assessment differ, but it seems likely that the public outcry at the thought that Breivik might not be detained in jail for the crimes he committed, and might be sentenced to compulsory treatment instead, influenced the course of events. Together with pressure from public opinion, other factors might have contributed to the need for a second assessment, including the fact that Breivik himself wanted to be held responsible for his actions and was unhappy about the diagnosis of schizophrenia and the prospect of compulsory treatment. We do not argue for a specific interpretation of the events, but want to discuss one assumption made by many participants in this debate, and by the Norwegian legal system itself, that having a certain set of psychiatric symptoms or a particular diagnosis (e.g., psychotic symptoms or schizophrenia as opposed to personality disorder) is sufficient by itself to determine whether there is moral and legal responsibility for (criminal) action. The Breivik case has highlighted the need to develop a more local and nuanced view of responsibility and of the kind of punishment that might be appropriate for criminal action. More precisely, an argument is needed to support the claim that the criteria used to discriminate schizophrenia from personality disorder (e.g., the presence of delusions) are also appropriate criteria for criminal insanity.

The assumption that people who have psychotic symptoms or have received a diagnosis of schizophrenia lack responsibility or have reduced responsibility for action is especially problematic, as the behaviour of two people with psychosis or schizophrenia can differ almost entirely. Some people with schizophrenia are able to function well, cognitively and socially, and to control their delusions to some extent. Given this clinical diversity, some authors (e.g., [7, 8]) have argued those who have the diagnosis of schizophrenia make up such a heterogeneous group empirically that the diagnosis is not a good guide for research. Should it be a good guide to determining responsibility for action? The presence of psychiatric symptoms and of a diagnosis of schizophrenia should be taken into account in the courtroom, but it should not be regarded as sufficient to determine responsibility.

## Delusional Beliefs

How can we establish whether a psychiatric condition impairs the making of a specific decision leading to criminal action? Neuroscience is making progress in identifying brain lesions which may impair or contribute to impairing normal decision-making, but the techniques developed so far are not sophisticated enough to allow inferences from present to past behaviour and thus cannot be used in arguing for or against the claim that a person was responsible for her action when she committed a criminal act.

It is beyond the data generated from any currently published scanning protocol to make predictions about the rational capacity (or lack thereof) of a criminal defendant, or to make inferences as to that defendant’s intent at a specific moment in time before or during a specific criminal act. ([9], page 41)

In the Breivik case, one key question for psychiatrists involved in his assessment was whether his system of beliefs was *delusional*. The presence of delusions alone would have indicated lack of responsibility according to the Norwegian Law. The psychiatrists who first assessed Breivik were struck by some of the claims he made, such as the claim that he was the ideological leader of a Knights Templar organisation. Breivik was convinced that this organisation existed and also reported to have attended its founding ceremony in London in 2002. Other bizarre beliefs he reported were that he would soon become the new regent of Norway, and that he could decide who was to live and die in the nation. Given the content of such beliefs, he was originally thought to have grandiose delusions. Although his anti-Islamic and more generally racist views were shared by others in some of the extremist groups he associated with, beliefs about his specific role in present and future cleansing projects seemed to be more markedly delusional and idiosyncratic.

Examining the social context of a person’s beliefs is important in a diagnostic setting as it is part of the definition of delusions in the DSM that they are not shared by the person’s community or sub-community. Is the social context of a person’s beliefs also relevant for the purposes of ascribing responsibility for action? For claims about responsibility, the significance of the fact that some of Breivik’s beliefs were not shared may derive from the following consideration. If poor reality testing (or some other relevant cognitive deficit associated with delusion formation) is affecting the beliefs he is prepared to endorse to the extent that such beliefs are implausible even to members of groups that are inclined to share his political and ideological views, then maybe such failure of reality testing (or other relevant cognitive deficit) is also implicated in some of his decision making processes, including those processes that led him to his criminal acts. But this is just a hypothesis that needs to be tested.

What we know about Breivik is that both the racist and anti-Islamic beliefs held by extremist groups and his own beliefs about his role in cleansing Norway from undesirable people are *epistemically bad*, because they are badly supported by evidence and insulated from counter-evidence. They are also *potentially dangerous*, because given their content they have the potential to lead to decisions and actions that will cause harm to others. Breivik had *mundane* racist and anti-Islamic beliefs and, in addition to those, *idiosyncratic* beliefs about a leading role in the cleansing project he was committed to. In terms of providing some rationale for his actions, both types of beliefs seem to be relevant to his criminal actions, in the sense that we can see such actions not as random acts of violence, but as consistent with his belief system. The presence of delusional beliefs as opposed to false, unjustified, and dangerous beliefs does not seem to carry any special weight unless it also signals the presence of some specific deficit in decision making.

One further point is that, even if we combine them, Breivik’s mundane racist beliefs and his more idiosyncratic grandiose beliefs do not seem to provide sufficient motivation for his actions. Breivik could have genuinely believed that multiculturalism significantly harmed Norwegian society (as many of the members of extremist groups do) and that he was in charge of an organisation fighting multiculturalism, without engaging in the actions that led him to kill 77 people. Those thoughts could have been channelled into joining a political party in which such views were shared or campaigning against Muslims and multiculturalism. In order to motivate murder, some other beliefs needed to be in place, and attention should be drawn to those, whether of a delusional nature or not. In this respect, we want to draw a parallel with the case of a young man with a diagnosis of schizophrenia who attacked his neighbour after experiencing auditory hallucinations about the neighbour making loud noise and insulting him repeatedly.

[S]uppose Bill had actually had a very noisy neighbor. What kind of ascription of responsibility would we have made in relation to the harm inflicted on his neighbor in those circumstances? What kind of punishment would Bill have deserved for his attacking his truly noisy neighbor? Should the fact that the experiences were hallucinatory (and thereby that the neighbor was not in fact noisy) make a difference in relation to how we conceive of Bill’s responsibility for what he did and of the punishment he deserves? It is true that Bill was hallucinating: He was hallucinating that his neighbour was making loud noises, and the content of the hallucination explains in part why he attacked his neighbor. Had he not hallucinated that his neighbor was making loud noises, Bill would have probably not attacked and harmed his neighbor. But it is also true that having noisy neighbors does not morally justify assaulting them. That is, had Bill’s neighbor been truly noisy, Bill would have still been doing something blameable in assaulting his neighbor. If one has a noisy neighbor, then one should try to convince his neighbor to be less noisy, and, failing that, one should perhaps call the police. ([10], page 182)

Here, what we find is that the psychotic symptoms experienced by Bill help explain his aggressive behaviour towards his neighbour, although they are not sufficient to motivate his actions. We believe the same type of consideration could be applied to Breivik and other mass murderers. They have beliefs that help explain why they committed horrendous crimes, but such beliefs alone should not be regarded as sole determinants for their actions — their actions are not inevitable or excusable given their beliefs.

This seems to have consequences for the relationship between delusions and criminal responsibility. The presence of delusions that help explain why one committed a crime is not sufficient to regard the person who committed the crime unaccountable due to insanity, though of course the presence of delusions is relevant to the person’s full psychological profile at the time the crime was committed and thus should be taken into account.

## Conclusions

We raised some preliminary concerns about the very direct connection (openly acknowledged in Norwegian law and more implicit but still influential in English law) between having a set of symptoms (e.g., psychotic symptoms) or having a certain psychiatric diagnosis (e.g., schizophrenia) and being held unaccountable for one’s actions. People with the same diagnosis may behave in very different ways and further information about individual cases is required – especially information about how impairments associated with one’s mental illness may affect decision making more generally, and specifically the making of the decisions which led one to commit the crime.

Moreover, we made two suggestions about the presence of delusions, which is often considered as a key criterion for criminal insanity. First, in terms of how delusions motivate criminal action, the role of delusional beliefs does not seem to be different from the role of non-delusional beliefs, unless we assume that the presence of delusions also signals the presence of a cognitive deficit that impacts on the decision to commit the crime in question (and at present it would be difficult to find empirical support for such a hypothesis). Second, having beliefs that are epistemically bad and potentially dangerous, whether delusional or not, is not always sufficient to motivate criminal action. It may contribute to an explanation for the crime, but does not make the criminal action inevitable or excusable. Thus, the presence of delusions seems to be neither necessary nor sufficient for criminal insanity.

Finally, reflection on the Breivik case and other similar cases should promote the development of a more nuanced view of responsibility for action, and of the relationship between psychiatric symptoms (often as part of a diagnosis) and criminal insanity. Such a notion of responsibility would have countless benefits: it would be theoretically stronger and better supported by the available evidence, and it would help us challenge the two misconceptions that Wessely described, that “outrageous crimes must mean mental illness” and that “the purpose of psychiatry is to get people off”. These simplistic conceptions are unfair to the mentally ill, who are stigmatised as a result, and to psychiatrists, whose clinical assessments and expert opinions are subject to significant political pressures when high profile cases come to the public attention.
